# A New Axial Stress Measurement Method for High-Strength Short Bolts Based on Stress-Dependent Scattering Effect and Energy Attenuation Coefficient

**DOI:** 10.3390/s22134692

**Published:** 2022-06-22

**Authors:** Tong Fu, Ping Chen, Aijun Yin

**Affiliations:** College of Mechanical Engineering, Chongqing University, Chongqing 400044, China; fifaft@163.com (T.F.); aijun.yin@cqu.edu.cn (A.Y.)

**Keywords:** bolt axial stress, acoustoelastic effect, scattering attenuation, stress-dependent attenuation coefficient, sensitive frequency band

## Abstract

The accurate estimation of axial stresses is a major problem for high-strength bolted connections that needs to be overcome to improve the assembly quality and safety of aviation structures. However, the conventional acoustoelastic effect based on velocity-stress dependence is very weak for short bolts, which leads to large estimation errors. In this article, the effect of axial stress on ultrasonic scattering attenuation is investigated by calculating the change in the energy attenuation coefficient of ultrasonic echoes after applying axial preload. Based on this effect, a stress-dependent attenuation estimation model is developed to measure the bolt axial stress. In addition, the spectrum of the first and second round-trip echoes is divided into several frequency bands to calculate the energy attenuation coefficients, which are used to select the frequency band sensitive to the axial stress changes. Finally, the estimation model between axial stress and energy attenuation coefficients in the sensitive frequency band is established under 20 steps of axial preloads. The experimental results show that the energy attenuation coefficient in the sensitive band corresponds well with axial stress. The average relative error of the predicted axial stress is 6.28%, which is better than that of the conventional acoustoelastic effect method. Therefore, the proposed approach can be used as an effective method to measure the axial stress of short bolts in the assembly of high-strength connections.

## 1. Introduction

The aviation structure contains many precise bolt connection structures, and a loose bolt may lead to abnormal mechanical vibration or even local disintegration, thus causing serious aviation safety accidents. Controlling the magnitude of the axial force of the bolt is an important measure to improve the connection quality of the aviation structure and reduce the incidence of aviation accidents. As the aviation structure has high speed and high vibration characteristics, it is necessary to strengthen the accurate measurement of the bolt axial stress in the aviation structure to make the connection stable without breaking the ring and then improve the overall connection level. Therefore, accurate prediction of axial stresses and control of bolt preload is important to ensure machine functioning and structural stability. There are several methods for estimating axial stress and controlling bolt preload, including the torque method [1,2], the strain gauge method [3,4], and the ultrasonic method [5]. The torque method generates the expected tightening torque through manual torque wrenches or pneumatic, hydraulic wrenches to control the bolt preload, which depends on the fact that the torque applied to the bolt can be effectively converted into the preload of the bolt. However, only about 10–15% of the torque can be converted into axial preload due to the different friction coefficients between the bearing surfaces of the thread and the nut. Some reports have shown that the error in axial stress measurements based on the torque method exceeds 30%. The strain gauge method uses the strain in the surface of the bolt to obtain its axial stress. This method is limited by the size of the measurement conditions, which makes it difficult to install the strain gauge on the stressed part of the bolt. The ultrasonic method has the advantages of high adaptability, accuracy, and stability, and this is the main development direction of axial stress measurement in the future.

Ultrasonic non-destructive methods, such as the piezoelectric ultrasonic method [6,7,8], the EMAT ultrasonic method [9,10,11], and the acoustoelastic effect method [12,13,14,15,16,17], have been reported to measure bolt axial stress. The piezoelectric ultrasonic method is primarily used for loosening the monitoring of bolted connections. Recently, the smart piezoelectric bolt is developed by embedding a piezoceramic transducer in the bolt head to improve measurement accuracy [18,19]. However, the devices used in this method are costly and complicated to install. As far as the EMAT method is concerned, a high-frequency generator is required. Furthermore, the response signal of the EMAT is easily disturbed by noise [20]. Among these methods, the acoustoelastic effect method is the most widely used. By adopting the acoustoelastic effect, ultrasonic longitudinal and transversal velocities can be used to characterize axial stress [15]. Nohyu Kim [12] used the velocity ratio of the mode conversion wave propagated in the bolt to estimate the axial stress of high-strength bolts. To improve the measurement accuracy, Yongmeng Liu et al. [13] proposed a method of measuring the fastening force with dry coupling. Dry coupling is used instead of liquid coupling to improve coupling conditions affected by the bolt surface. Qinxue Pan et al. [17] focused on the non-uniform distribution of axial stress in the effective stressed region of the bolt. The shape factor is proposed to eliminate the impact of the stress distribution on the propagation path of ultrasonic waves on the measurement. YASUI et al. [16] developed a calibration method based on the longitudinal and transverse waves for the short bolt. However, it needs to establish a large number of calibration curves to compensate for the difference in stress conditions in the actual stress measurement. Enxiao Liu et al. [14] investigated the influence of coupling layer thickness on the bolt axial stress. The result shows that the influence of the thickness change of the coupling layer on the accuracy of axial stress measurement is greater for short bolts than for long bolts. Apparently, the results of these methods are highly dependent on the accuracy of the time of flight (ToF) measurements, which can be significantly influenced by the bolt length. The principle of the conventional acoustoelastic effect method is to determine the axial stress by the difference in wave propagation time. Both the ultrasonic signal before and after bolt tightening should be measured precisely to obtain an accurate ToF. For the short bolts, the absolute axial elongation under the same load will be reduced accordingly, so that the difference in propagation time before and after applying axial preload will become difficult to distinguish, leading to invalid or large error measurement results. In this case, the acquisition of ToF is a great challenge for the employed AD digitizer. Therefore, the conventional acoustoelastic effect method is not ideal for the axial stress evaluation of short bolts.

In recent decades, ultrasonic scattering theory based on the interaction of microstructure with waves has been greatly developed, making attenuation-based methods an effective nondestructive evaluation tool. It has been widely used to extract microstructural parameters such as grain size, boundary, and dislocation [21,22,23]. In research on the ultrasonic attenuation method, the mechanism of scattering attenuation is highlighted [24,25,26,27,28]. Recently, Turner and Ghoshal [24] presented a theoretical basis to extract stress information from polycrystalline microstructure based on ultrasonic scattering attenuation. It was later extended and generalized by Kube [26]. The covariance tensor of elastic modulus variations was included as part of the previously developed ultrasonic grain scattering models and is proportional to the attenuation and backscatter coefficients [25,27,29]. Kube et al. [28] confirmed the stress dependence of the covariance tensor by investigating the change of the spatial variance amplitude under an applied uniaxial load. The results show that attenuation-stress variations are two to four times larger than the wave velocity-stress variations for metallic and molecular bonding materials. Therefore, the stress-dependent attenuation effect is more suitable for high-strength short bolts that are not sensitive to the conventional acoustoelastic effect.

The stress-dependent scattering effect is based on the change in the attenuation of acoustic energy when ultrasound waves propagate through materials subjected to different uniaxial stresses. The relationship between stress and attenuation coefficient can be modeled with the help of the attenuation coefficient spectrum. Furthermore, the attenuation coefficient spectrum provides a detailed analysis of attenuation in the frequency domain, which can comprehensively show the relationship between stress, attenuation coefficient, and frequency. However, the main challenge is to obtain reliable frequency-dependent attenuation coefficients. In the traditional attenuation coefficient measurement method, the center frequency of the ultrasonic echo signal may shift downward due to the scattering attenuation effect of the material grain boundary, making it difficult to select a reasonable effective bandwidth range for further research [30], and the peak frequency and peak value only reflect the fundamental frequency components of the ultrasonic echo signal; the corresponding energy amount is not sufficient to reflect all the characteristics of the ultrasonic echo. Xiongbing Li et al. [31] established a multi-scale ultrasonic attenuation evaluation model based on wavelet transform, which uses attenuation coefficients at different scales to effectively control the errors arising from the fitting of the model itself. In addition, Min Li et al. [32] discovered that the frequency bands are sensitive to grain size variations based on the energy attenuation coefficient spectrum. The results show that the evaluation modes built using the energy attenuation coefficients of the sensitive bands have higher accuracy compared to other bands. Gao et al. [33] proposed a frequency bandwidth selection rule for amplitude ratio linear regression to determine the attenuation spectrum and they proposed a method to further improve the stability and accuracy. Based on the current research introduced above, the extraction of the spectral characteristic parameters confirms the fact that the ultrasonic scattering attenuation is sensitive to the frequency band, which means that the properties of the material make it attenuate differently in different frequency bands.

In this study, a novel bolt axial stress measurement method based on the stress-dependent attenuation effect and energy attenuation coefficient is developed. The bandwidth of the measured echoes is divided into several frequency bands to select the frequency band sensitive to the axial stress changes. This article is organized as follows. First, the important concepts of stress-dependent elastic wave scattering theory are introduced. Second, the estimation model of axial stress on ultrasonic scattering attenuation is derived. The process of the calculation of the energy attenuation coefficient and the selection of the sensitive frequency band is presented. Following that, a series of experiments are then implemented to validate the proposed method. The results of axial stress measurements with different frequency bands and clamping lengths are analyzed. Furthermore, a comparison with the conventional acoustoelastic effect method was conducted to verify the accuracy and stability of the proposed method.

## 2. Ultrasonic Measurement Model of Bolt Axial Stress Based on the Energy Attenuation Coefficient

### 2.1. Stress-Dependent Attenuation Theory

In the models of Turner and Ghoshal [24] and Kube [26], the acoustoelastic of single crystals was derived by considering polycrystals as an ensemble of crystals and deriving the associated tensor of the effective stress-dependent elastic modulus. The stress-independent covariance tensor is defined as:

(1) $$\Xi_{ijkl}^{\alpha \beta \gamma \delta }=\langle C_{{}_{ijkl}}^{*}C_{\alpha \beta \gamma \delta }^{*}\rangle -\langle C_{{}_{ijkl}}^{*}\rangle \langle C_{\alpha \beta \gamma \delta }^{*}\rangle$$

where $C_{ijkl}^{*}$ is the stress-dependent effective elastic modulus tensor of the polycrystalline materials. 〈 〉 denote the ensemble average moduli. $\langle C_{ijkl}^{*}C_{\alpha \beta \gamma \delta }^{*}\rangle$ is performed as an average over all possible crystal orientations. For a homogeneous single crystal, it can be written as $G_{ijkl}=C_{ijkl}+\left(-C_{ijkl}S_{rrpq}+C_{ijkl}S_{rlpq}+C_{ijrl}S_{rkpq}+C_{irkl}S_{rjpq}+C_{rjkl}S_{ripq}+C_{ijklmn}S_{nmpq}\right)\sigma_{pq}$, where $C_{ijkl}$, $C_{ijklmn}$ are the second-order elastic modulus tensor and third order elastic modulus tensor, $S_{ijkl}=C_{ijkl}^{-1}$ is the elastic compliance tensor, and $\sigma_{pq}$ is the stress tensor. For crystallites of cubic symmetry, the third-order elastic modulus tensor can be written as [24]

(2) $$C_{ijkl}=C_{ijkl}^{I}+v\sum_{n=1}^{3}a_{in}a_{jn}a_{kn}a_{ln}$$

where $\nu =c_{11}-c_{12}-2c_{44}\;$ is the anisotropy coefficient, and $C_{ijkl}^{I}$ is the isotropic fourth-rank tensor. $a_{ij}$ is rotation matrices with respect to the rotating coordinate system.

According to Equations (1) and (2), Kube derived a relationship between the attenuation coefficients and the anisotropy third-order elastic constants. The stress-dependent attenuation coefficient is defined as the scattered energy lost from the wave with polarization I scatter into polarization p [28]:

(3) $$\alpha_{I\to P}=\frac{{\left(wl\right)}^{4}}{2\rho^{2}V_{I}^{3}V_{P}^{5}}\times \int_{-1}^{1}\frac{h\cdot \Gamma^{I\to S}\cdot g^{T}}{{\left[1+{\left(wl\right)}^{2}\left(V_{I}^{-2}+V_{P}^{-2}-2V_{I}^{-1}V_{P}^{-1}\cos\theta \right)\right]}^{2}}\times d(\cos\theta )$$

which is a general expression for the longitudinal and shear wave modes. *w* is the wave angular frequency, *l* is the mean diameter of the grains, *ρ* is the density of the material, and $V_{I}$ and $V_{P}$ are the velocities of the polarization I and the polarization *P*, respectively. *θ* is the scattering angle between the incident wave vector and I→*P* denotes the wave with polarization I scatters into polarization *P*. **h** = [1 T T^2^] and **g** = [1 cos^2^*θ* cos^4^*θ*] are the row-vectors of the stress magnitude and the scattering angle. The term $\Gamma^{I\to S}$ in Equation (3) is a 3 × 3 matrix for the different possible scattering modes. For cubic symmetry crystallites, the total longitudinal matrices are:

(4) $$\begin{array}{l} \Gamma^{L\to L}=\left[\begin{array}{ccc} 9 & 6 & 1\\ -9\zeta & -6\zeta & -\zeta \\ -9\zeta^{2}/4 & 3\zeta^{2}/2 & \zeta^{2}/4 \end{array}\right]\\ \Gamma^{L\to SH}=\left[\begin{array}{ccc} 5 & 5 & 0\\ -5\zeta & -\zeta & 0\\ 5\zeta^{2}/2 & \zeta^{2}/4 & -\zeta^{2}/4 \end{array}\right]\\ \Gamma^{L\to SV}=\left[\begin{array}{ccc} 10 & 1 & -1\\ -10\zeta & -\zeta & \zeta \\ 5\zeta^{2}/2 & \zeta^{2}/4 & -\zeta^{2}/4 \end{array}\right] \end{array}$$

where ξ=2(ν+η)(3ɢν)  is a parameter related to the anisotropy constants and bulk modulus. $ɢ=\left(c_{11}+c_{12}\right)/3$ is the bulk modulus of the cubic crystallite and $\nu =c_{11}-c_{12}-2c_{44}$ and $\;\eta =c_{111}-c_{123}-2c_{144}-4c_{155}$ are second and third-order elastic anisotropy constants for cubic crystals, respectively. This assumes that the spatial correlation function η(r)=exp(−r/l) that described the two random points separated by the distance *r* is generally linear in the same grain. The total longitudinal attenuation constants are then:

(5) $$\alpha_{L}=\alpha_{L\to L}+\alpha_{L\to SH}+\alpha_{L\to SV}$$

The subscripts $\alpha_{L\to L},\;\alpha_{L\to SH\;}$ and $\;\alpha_{L\to SV}$ represent the three scattering conversion modes of the incident longitudinal waves, respectively. Finally, in the Rayleigh scattering attenuation zone ($wl^{2}/V_{L}^{2}$)≪1, the integration in Equation (3) for the longitudinal wave attenuation coefficient can be expressed as

(6) $$\alpha_{T}^{L}=\frac{{\left(2\pi f\ell \right)}^{4}\nu^{2}}{375\rho^{2}V_{L}^{8}}\left(8+12\frac{V_{L}^{5}}{V_{S}^{5}}\right){\left(1-\frac{\zeta }{2}T\right)}^{2}$$

where *V_L_*, and *V_s_* are the velocities of the longitudinal wave and the shear wave, respectively. Equation (6) builds a connection between the magnitude of the stress *T* and the strength of the ultrasonic attenuation. It should be noted that the formula is valid only under the condition that stress is not causing the polycrystal to yield.

### 2.2. The Axial Stress Measurement Model Based on the Stress-Dependent Attenuation

**Figure 1:**
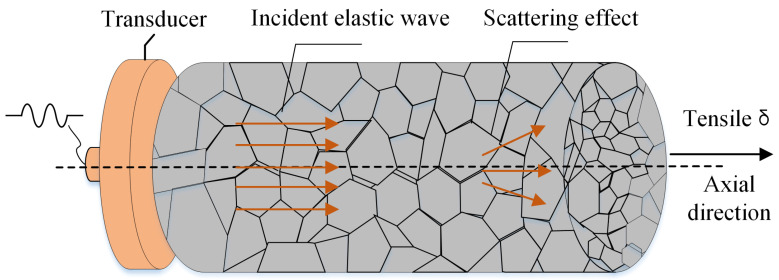
Ultrasonic wave propagation in bolt under the axial stress.

When an ultrasonic wave propagates through the polycrystalline material, its energy attenuates due to wave diffraction, scattering, and absorption by the interaction between the waves and the grains. In ultrasonic testing, the scattering-induced attenuation is the loss due to the perturbation of ultrasonic waves when the acoustic impedance between heterogeneous media does not match the interface (grain boundaries). Because polycrystalline materials are usually not viscoelastic, the absorption attenuation can be negligible in our study. In addition, the sidewalls of the rod component can suppress the diffusion of the acoustic beam by reflecting the dissipated energy. Therefore, in the bolt specimen, the attenuation of ultrasonic waves is essentially produced by the scattering process. To illustrate the dependency between stress and ultrasonic attenuation, a stress-free polycrystalline cylindrical specimen (with cubic crystal symmetry) subjected to tensile preload δ in the axial direction is considered, as shown in Figure 1. Based on the unified scattering theory [21], the attenuation of ultrasound waves in the MHz range propagating in polycrystals strongly depends on the scattering occurring at the grain boundaries. Due to the stress dependence of crystallites in their effective elastic characteristics, the attenuation coefficient, which results in the scaling of the wave amplitude during propagation, carries information about the stress and frequency properties.

**Figure 2:**
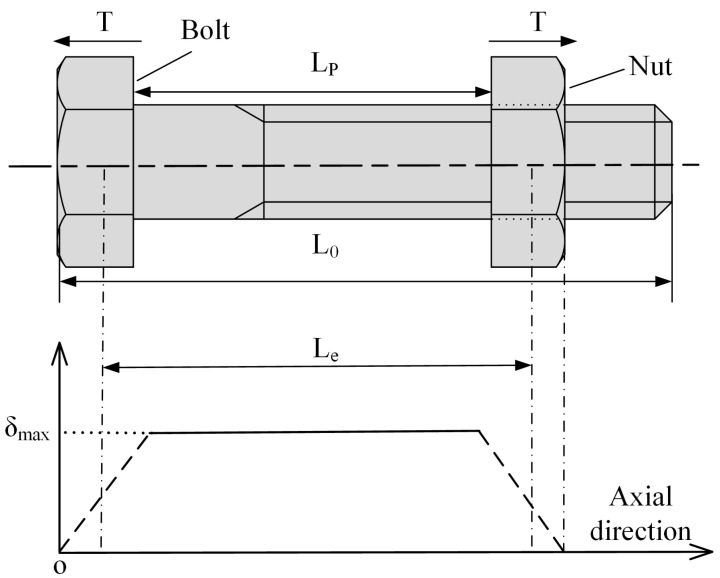
The one-dimensional axial stress model.

We consider a one-dimensional model to calculate the response of ultrasonic waves to axial preload, and the axial stress distribution over the length of the bolt is shown in Figure 2. The stresses in the middle of the bolt are approximately uniform, whereas the stresses near the head and threads show a gradient pattern along the axial direction [17]. To simplify the problem, we assume that the full bolt length is the sum of the equivalent stressed length and the unstressed portion. The entire stressed part of the bolt is equivalent to a cylinder with uniformly distributed stresses, the length of which is defined as the equivalent stressed length. In Figure 2, *L*_0_ is the full length of the bolt, *L_e_* is the equivalent stressed length, and *L_p_* is the clamping length. Therefore, the attenuation coefficient of the bolt under axial preload can be expressed as:

(7) $${\bar{\alpha }}_{T}=\frac{L_{e}}{L_{0}}\alpha (\delta )+\frac{L_{0}-L_{e}}{L_{0}}\alpha (0)$$

where α(0) and α(δ) are the attenuation coefficients of the stress-free and equivalent stressed sections of the bolt. It should be noted that the effective stressed length *L_e_* is dependent on the axial preload, which means that *L_e_* will vary as the axial stress changes. Because the clamping length *L_p_* is found to be linearly related to *L_e_* [17], the effective length ratio *β* can be used as a substitute for *L_e_*/*L*_0_. Substituting (6) into (7):

(8) $$\begin{array}{l} {\bar{\alpha }}_{T}=\frac{\beta {\left(2\pi fl\right)}^{4}v^{2}}{375\rho^{2}V_{L}^{8}}\left(8+12\frac{V_{L}^{5}}{V_{S}^{5}}\right){\left(1+\frac{\zeta }{2}T\right)}^{2}+\frac{(1-\beta ){\left(2\pi fl\right)}^{4}v^{2}}{375\rho^{2}V_{L}^{8}}\left(8+12\frac{V_{L}^{5}}{V_{S}^{5}}\right) \end{array}$$

The effective length ratio *β* is expressed as

(9) $$\beta =\frac{kL_{p}+b}{L_{0}}$$

where *k* and *b* are the coefficients with respect to the clamping length and axial preload. Equation (8) can be further simplified as follows:

(10) $${\bar{\alpha }}_{T}^{}=\beta A\left[{\left(1-\frac{\xi }{2}T\right)}^{2}-1\right]+A=\beta A\frac{\xi^{2}}{4}T^{2}-\beta A\xi T+A$$

(11) $$A=\frac{{\left(2\pi fl\right)}^{4}v^{2}}{375\rho^{2}V_{L}^{8}}\left(8+12\frac{V_{L}^{5}}{V_{S}^{5}}\right)$$

where *A* is a coefficient for the model associated with material parameters *ρ*, *ν*, *V_L_*, *V_S_*, and *f* (the frequency in the employed frequency band). It can be seen that *A*, *β*, and ζ are stress-dependent constants related to the applied stress preload and frequency. Equation (10) builds a connection between the magnitude of the bolt axial stress *T* and the attenuation coefficient.

*ζ* is negative for almost all metals [28]. Several observations can be derived from Equation (10). First, the attenuation coefficient ${\bar{\alpha }}_{T}$ will increase with the tensile preload. Secondly, *β* is positively correlated with the attenuation coefficient ${\bar{\alpha }}_{T}$ under the same stress. Finally, because the ratio of *ζ* to *T* is large (*ζ*/*T* > 10^3^ MPa), the scattering response is expected to be nearly linear for low stress (<500 MPa).

### 2.3. Energy Attenuation Coefficients

**Figure 3:**
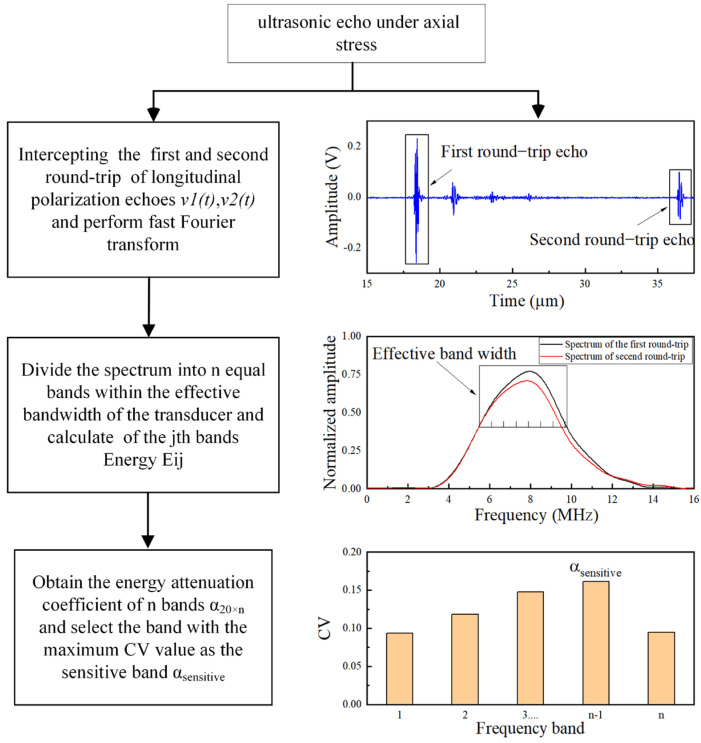
Flow chart of the calculation of energy attenuation coefficient.

As discussed in the introduction, the main problem in establishing an estimation model using Equation (10) is to obtain a reliable frequency-dependent attenuation coefficient. On the one hand, it is desired to obtain the attenuation coefficient that can effectively reflect the relationship between attenuation and stress. On the other hand, it is desired to know whether the attenuation coefficients can better distinguish the change of axial stress in a specific frequency range. As shown in Figure 3, to model the relationship between the stress, attenuation, and frequency, the frequency spectrum energy is proposed to calculate the attenuation coefficient, and the calculation is performed in multiple frequency bands to find what is sensitive to axial stress changes. The detailed process is described as follows.

During the propagation process, the ultrasonic waves will occur in a series of reflections and mode conversions at the bolt boundary. When the ultrasonic longitudinal transducer is located at the surface of the bolt head, part of the signal in the main flap region is unaffected by the boundary and reaches the end of the bolt directly. Therefore, the normally incident longitudinal-to-longitudinal ultrasound (travels one and two round-trips in the bolt of longitudinal wave polarization direction) is used to characterize scattering attenuation under uniaxial stress, as shown in Figure 3. A rectangular window is employed to intercept ultrasonic echoes traveling the first round-trip $v_{1}(t)$ and second $v_{2}(t)$ round-trip of the longitudinal polarization direction and the echoes of the time domain are converted to the frequency domain, which can be expressed as:

(12) $$S_{i}(f)=\int_{t_{1}}^{t_{2}}v_{i}(t)e^{-j2\pi ft}dt$$

where *i* represents *i*_th_ round-trip echo, *i* = 1, 2. Then, the frequency spectrum is divided into n bands within the effective bandwidth of the transducer, and each band has an equal bandwidth. The frequency spectrum of each band is $\Delta \left|S_{i,j}\left(f\right)\right|$, *j* represents the *j*_th_ frequency band, *j* = 1,2 …, n. It should be noted that n should be determined by the corresponding effective frequency bandwidth, and a high value of n does not necessarily achieve good results.

The spectrum energy *E_i;j_*, by integrating each band $\Delta \left|S_{i,j}\left(f\right)\right|$ is expressed as:

(13) $$E_{i,j}=\int_{f_{j}^{b}}^{f_{j}^{t}}(\Delta \left|S_{i,j}(f)\right|{)}^{2}df$$

where $f_{j}^{t}\;$ and $f_{j}^{b}$ represent the top and bottom limits of each frequency band, respectively.

The spectrum energies of the *j*_th_ frequency band of the first round-trip and second round-trip echoes, respectively, are obtained as $E_{1,j}$ and $E_{2,j}$. Then, the energy attenuation coefficient of the *j*_th_ frequency band can be expressed as:

(14) $$\alpha_{j}=\frac{10}{L_{0}}\ln\left(E_{1,j}/E_{2,j}\right)$$

where *L*_0_ is the length of the bolt. The energy attenuation coefficient in the n frequency band, *α*_1_, … *α_j_*, … *α_n_* can be obtained, which can reflect the relationship between attenuation coefficient *α* and frequency *f*.

In this study, 20 steps of axial preload were applied to the bolt and the signals collected under each axial preload step were converted into the frequency spectrum. Each frequency spectrum was then divided into n equal parts in the effective frequency band to obtain the energy attenuation coefficient matrix $\alpha_{20\times n}$. One row of the matrix corresponds to the data under one axial preload step. Each row contains energy attenuation coefficients for n frequency bands. One column of the matrix contains the energy attenuation coefficients for 20 steps of axial preload in the same frequency band, denoted by $\alpha_{j}$. If $\alpha_{j}$ exhibits obvious fluctuations compared to other frequency bands, then the energy attenuation coefficient in the *j*_th_ frequency band is more sensitive to axial stress change. This frequency band is called the sensitive band and is denoted by $\alpha_{sensitive}$. To find the sensitive band, the coefficient of variation (CV) was employed to analyze the fluctuations of the energy attenuation coefficient matrix in n frequency bands [32].

(15) $$\mathrm{CV}=\sigma_{\alpha }/\mu_{\alpha }$$

where $\sigma_{\alpha }$ and $\mu_{\alpha }$ are the standard deviation and mean value of the energy attenuation coefficient in the *j*_th_ frequency band, respectively. A high CV value implies a large fluctuation in the energy attenuation coefficient. Consequently, the estimation model between the axial stress and energy attenuation coefficient in the sensitive frequency band can be established. It should be noted that the dependence of the stress in the attenuation model comes from the quadratic form with the coefficient *ξ* and also from the dependence of the attenuation on the frequency itself; thus, *ξ* is not an indicator of the sensitivity of the material to the stress. The choice of the sensitive band is based on the attenuation of the material in the effective band of the transducer.

## 3. Experimental Setup

### 3.1. Experimental Equipment

**Figure 4:**
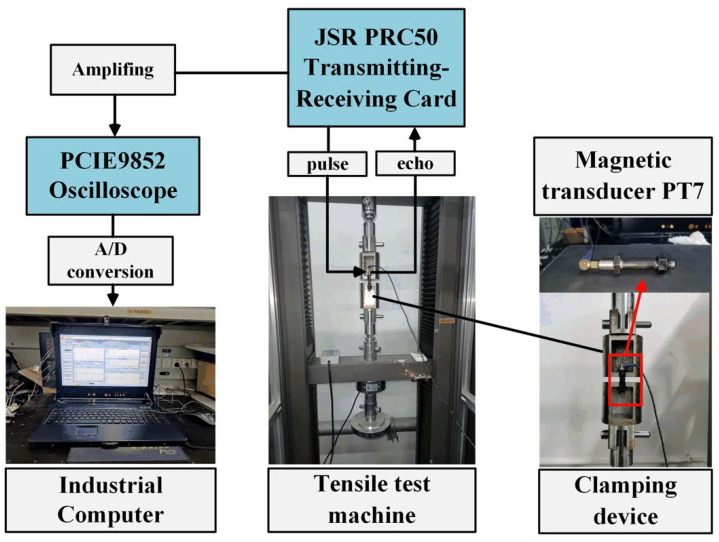
Schematic of the experimental configuration.

**Figure 5:**
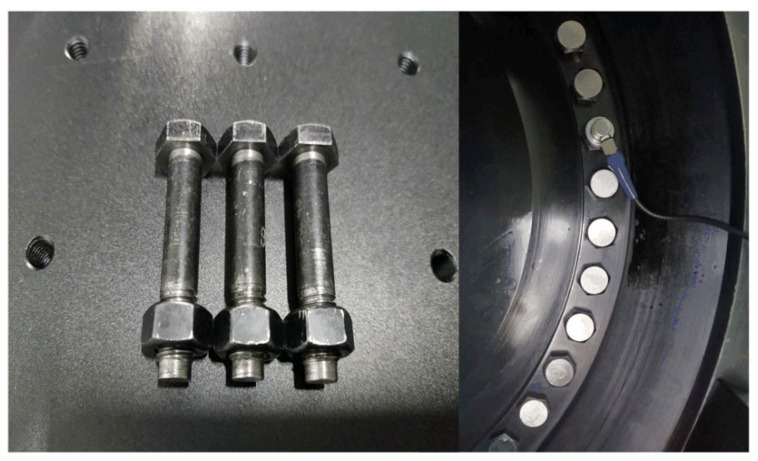
The polished surface of the bolt specimens.

The axial stress measurement experiment system is built as shown in Figure 4. The ultrasonic transmitting and receiving card JSR PRC50 (produced by JSR Ultrasonics) with a gain range from 14 dB to 60 dB is used to generate a high-voltage spike to excite the ultrasonic transducers. The AD-link PCIe-9852 oscilloscope (produced by AD-link Technologies) with a sampling rate of up to 200 MHz is selected as the data acquisition hardware. They are integrated into a portable industrial computer to form a DAQ system. To ensure stable coupling, the magnetic transducer PT7 (produced by the Dakota Ultrasonics of USA) with a 6 mm piezoelectric disc is used, and its parameters are shown in Table 1. The magnetic force brings the transducer in close contact with the end face of the bolt. The couplant B2 (produced by the Olympus Corporation) is applied on the bolt head surface to reduce the wave reflection in the bolt–transducer interface; the coupling layer thickness remains uniform and thin. Two coarse thread hex head bolts are used, and their detailed parameters are provided in Table 2 and Table 3. To avoid echo distortion and improve the coupling condition, the end faces of each bolt were finished with a milling machine before the measurement experiments. Figure 5 shows the image of the polished surface from bolt specimens. The bolt is installed on the CTM2200s tensile testing machine, which is produced by Xieqiang Co. Ltd. of China, through a specially designed jig. The device will ensure that the bolt is applied with a uniaxial preload; the clamping fixture can provide a fixed force to ensure stable contact between the transducer and the bolt at each step.

In addition, because the temperature is an important factor affecting the accuracy of bolt stress measurements [28], a heater and a thermometer with a resolution of 0.1 °C (JXB312, Berrcom Ltd., China) were used to control and monitor the measurement environment temperature. To avoid the influence of temperature changes on material attenuation and acoustic velocity, the entire experimental setup is located in a laboratory with a stable ambient temperature within ±1 °C by central air conditioning during the stress measurement tests.

### 3.2. Experimental Processes

According to Equation (10), the model parameters need to be calibrated before axial stress measurement. The calibrated process based on the energy attenuation coefficient is as follows:

(a) Install the bolt and attached the magnetic transducer on the surface of the bolt head and tighten the fixture device to ensure stable coupling between the transducer and the bolt before applying axial preload.

(b) Apply preload to the bolt to gradually increase its axial stress, and collect the ultrasonic echo signal data under the corresponding axial stress.

(c) Calculate the energy attenuation coefficient matrix $\alpha_{m\times n}$ based on the first round-trip and second round-trip echo signals corresponding to each axial preload. Furthermore, obtain the energy attenuation coefficient in the sensitive frequency band $\alpha_{sensitive}$ by selecting the highest CV value in n frequency bands.

(d) Obtain the α–T curve from the axial stress and $\alpha_{sensitive}$ using a nonlinear least-squares fitting technique with Equation (10). Then, the calibrated parameters are determined.

After the calibration parameters are obtained, the axial stress can be measured. The measurement process is as follows:

(a) Tighten the bolt to produce axial stress $T_{mea}$ and collect ultrasonic echo signal data under this axial stress.

(b) Obtain the energy attenuation coefficient in the sensitive frequency band $\alpha_{mea}$.

(c) Substitute the energy attenuation coefficient $\;\alpha_{mea}$ into the estimation model based on Equation (10) to obtain the axial stress.

**Figure 6:**
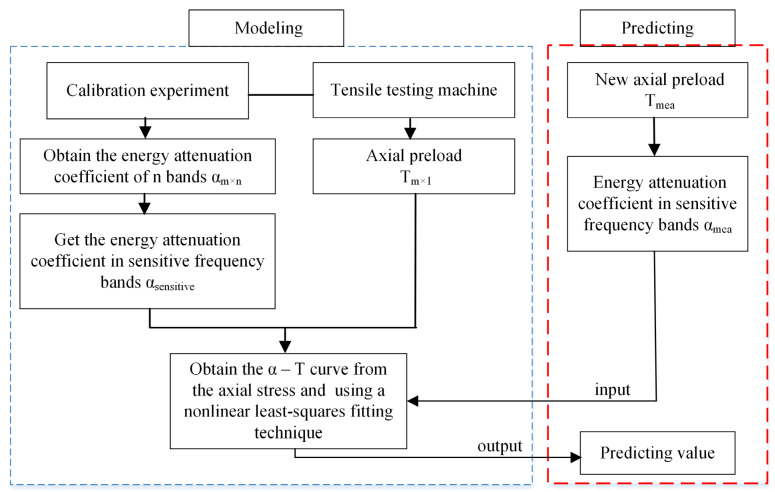
Modeling and predicting process.

A flow chart of these steps is shown in Figure 6.

## 4. Analysis of Experimental Results

To verify the validity of the proposed method, the following experiments were conducted. First, the bolt axial preload process was divided into 20 steps; each step of the tensile testing machine is set as 10 MPa, the range of loading is 0–200 MPa, and ultrasonic signals after each step were measured. Next, the energy attenuation coefficient matrix $\alpha_{20\times n}$ of the 20 steps was obtained to select the sensitive frequency band. The estimation model was built using the calibrated parameters. Thereafter, the results of other frequency bands and different clamping lengths were analyzed. Finally, experiments based on the conventional acoustic elastic method were carried out to compare them with the proposed method.

### 4.1. Analysis of the Ultrasonic Frequency Spectrum

**Figure 7:**
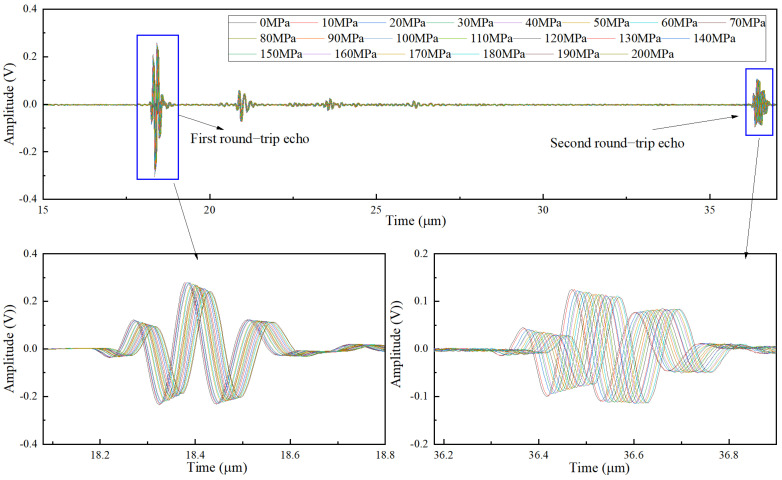
The echo signals under 20 steps of axial preload for the clamping length of 35.70 mm.

Figure 7 shows the changes of the first and second round-trip echo amplitude under 20 steps of axial preload for the clamping length of 35.70 mm. It can be clearly seen that the echo amplitude decreases with the increase in axial preload. This phenomenon indicates that the axial preload enhances the scattering effect and leads to more severe attenuation.

**Figure 8:**
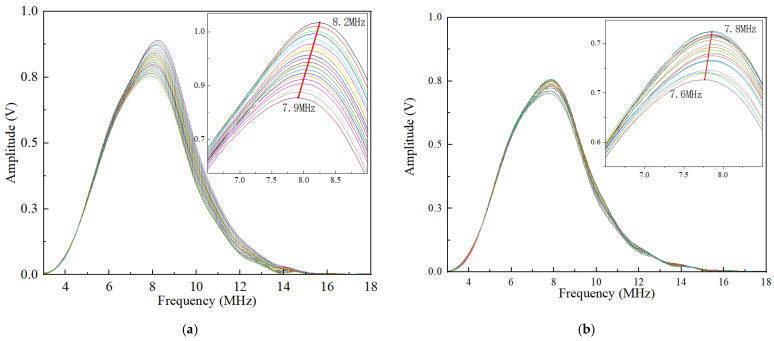
The frequency spectrum of the echo signals under 20 steps axial preload for clamping length of 35.70 mm: (**a**) frequency spectrum of the first round-trip echoes; (**b**) frequency spectrum of the second round-trip echoes.

Rectangular windows were used to extract the first and second round-trip echo signals, and then the fast Fourier transform (FFT) was used to analyze their frequency-domain characteristics. Figure 8a,b show the corresponding spectrum obtained from the FFT results. It can be seen that the center frequencies of the first and second round-trip echo decrease as the axial preload increases. The center frequency of the first round-trip echo was reduced from 8.2 to 7.9 MHz, and the second round-trip echo was reduced from 7.8 to 7.6 MHz, which is lower than the nominal value. This result is due to the fact that the attenuation of the high-frequency content is more severe than that of the low-frequency content, and the scattering attenuation effect is enhanced with the increase in the stress, which makes this phenomenon more obvious. In addition, it can also be found that the center frequency of the second round-trip echo is further reduced and moved to the left along the frequency spectrum compared to the first round-trip echo. The maximum center frequency of the first round-trip echo signal is close to 8.2 MHz. The center frequency of the second round-trip echo signal is further reduced with a maximum value of 7.8 MHz. The reason is that the second round-trip echo travels a longer distance and encounters more grain boundaries than the first round-trip echo.

It should be emphasized that the spectrum behavior of both the first and second round-trip echoes should be considered. From the frequency domain characteristics shown in Figure 8, the frequency range within the full width of the half-maximum of these first round-trip echoes is approximately 5.5–11 MHz, whereas the range of the second round-trip echo signal is approximately 5–10.5 MHz. Based on overall considerations, the range between 5.5 and 10.5 MHz was chosen as the effective bandwidth for calculating the energy attenuation coefficient.

### 4.2. Selection of Sensitive Frequency Bands

To improve the prediction accuracy of axial stress, it is necessary to select the sensitive frequency band energy attenuation coefficient to build the estimation model. The effective spectrum bandwidth in the range of 5.5–10.5 MHz is divided into five parts: 5.5–6.5 MHz, 6.5–7.5 MHz, 7.5–8.5 MHz, 8.5–9.5 MHz, and 9.5–10.5 MHz. The energy attenuation coefficients are calculated for each axial preload step in each frequency band. Thus, the energy attenuation coefficient matrix $\alpha_{20\times 5}$ can be obtained. One matrix row contains the energy attenuation coefficients of five frequency bands, and one matrix column contains the energy attenuation coefficients of 20 axial preload steps in the same frequency band.

**Figure 9:**
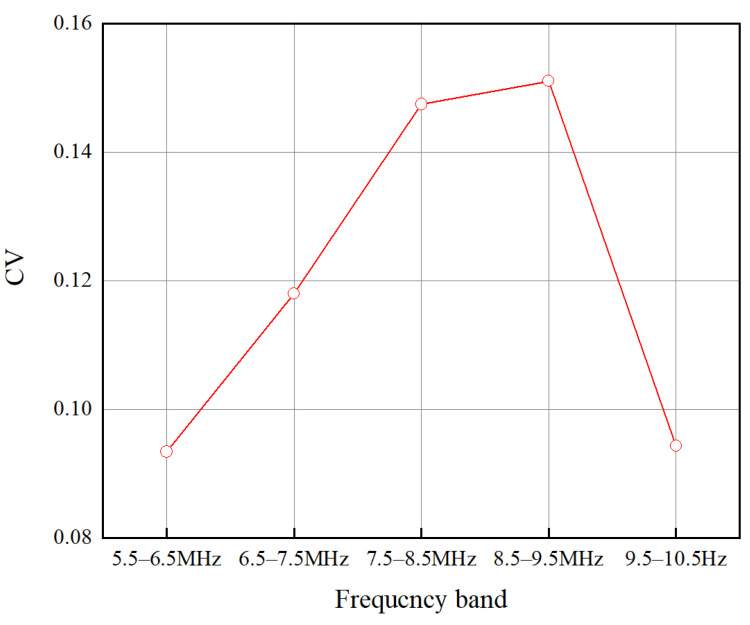
Value of CV in each frequency band.

The CV values for each band were calculated using Equation (15). The results are shown in Figure 9. It can be seen that the CV value of the energy attenuation coefficient in the 8.5–9.5 MHz band is the highest. This indicates that the fluctuation of the energy attenuation coefficient is the largest in this band and is highly sensitive to the stress-dependent attenuation changes. It is worth noting that the energy attenuation coefficient is calculated using the spectrum energy of the first and second round-trip echoes, which increases the CV value in the frequency band compared to the frequency peak-based calculation method. In this study, the frequency band of 8.5–9.5 MHz was chosen as the sensitive band to build the estimation model based on Equation (10).

**Figure 10:**
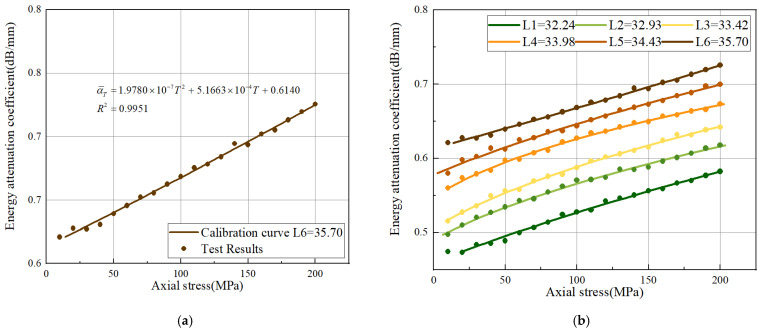
Fitting curves: (**a**) the calibrated curve of L6 = 35.70 in 8.5–9.5 MHz; (**b**) the calibrated curves of L1-L6 in 8.5–9.5 MHz.

The polynomial least squares fitting is used to model the measurement data. The correlation index $R^{2}$ was employed to evaluate the validity of the model in the modeling process. The range of $R^{2}$ is [0, 1]; a larger $R^{2}$ indicates a better approximation capability of the proposed model. Figure 10a shows the calibrated curve in the sensitive frequency band 8.5–9.5 MHz. The correlation coefficient $R^{2}$ of the estimation model is 0.9951. It is worth noting that the energy attenuation coefficient is positively correlated with the increase in axial stress.

In the proposed method, the calculation of the axial stress depends mainly on the energy attenuation coefficient of the ultrasonic echo in the stress region. According to Equation (10), the attenuation of ultrasonic echoes is also influenced by the clamping length, through the 20 steps of the axial preload calibrated process for six different clamping lengths. The relationship between clamping length and echo energy attenuation is illustrated in Figure 10b. It can be seen that the energy attenuation coefficient increases with the increase in the clamping length under the same axial preload. This is because the increasing clamping length will correspondingly increase the propagation distance of the ultrasonic echoes in the effective stress region, which enhances the scattering attenuation.

### 4.3. Measurement Results in Other Bands

In the process of axial stress estimation, the relative error is used to evaluate the accuracy of the prediction. A small relative error indicates the high accuracy of the prediction results. We further validate the effectiveness of the proposed method. Table 4 shows the prediction results for the sensitive frequency band and entire frequency band, and Table 5 shows the prediction results for the other four frequency bands.

The comparison between Table 4 and Table 5 shows that the minimum relative error in the 8.5–9.5 MHz band is only 2.35%. The relative errors for the other four frequency bands in Table 5 show large fluctuations. For example, in the results of the 8.5–9.5 MHz band, the relative error of 115.01 MPa is 2.70%. However, the relative errors of the 6.5–7.5 MHz band and 7.5–8.5 MHz band are both above 4.5%. Similarly, the relative errors of the prediction results based on all frequency bands are all larger than that of the sensitive frequency band. Furthermore, the correlation index *R*^2^ of 8.5–9.5 MHz is the closest to 1 of all the other bands.

**Figure 11:**
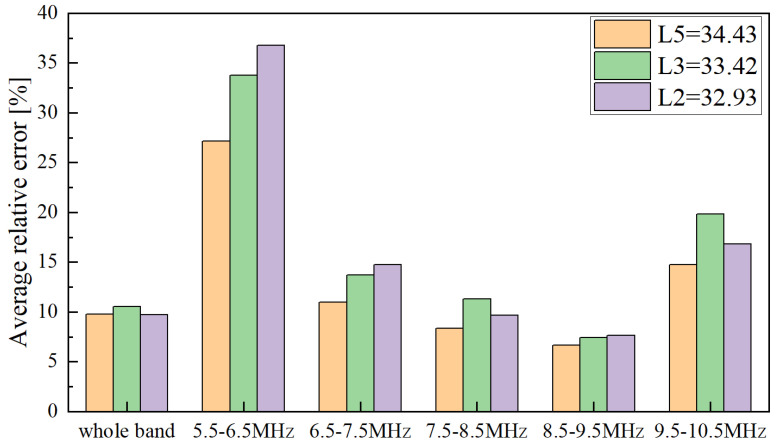
Average relative errors of three different clamping lengths.

Three clamping lengths of 34.43 mm, 33.42 mm, and 32.93 mm were selected to verify the sensitive frequency band. The actual axial preloads were re-selected, and eight axial stress measurements were carried out under each clamping length. The average relative errors for the five frequency bands and the entire band are shown in Figure 11. The results show that the average relative error in the 8.5~9.5 MHz band is also the smallest, which is approximately 7%. The average relative errors of other frequency bands are more than 9%. Therefore, selecting the energy attenuation coefficient in the sensitive band to build the estimation model is effective and necessary, which can better reflect the effect of axial stress on ultrasonic scattering attenuation.

### 4.4. Comparison of the Proposed Method and Conventional Acoustoelastic Method

Next, we tested the accuracy and repeatability of the proposed method in practical application. First, 20 steps of axial preload were performed with bolt A1 to build the estimation model. Second, 10 steps of axial preload were applied to bolt A2 and the energy attenuation coefficient in the sensitive band was calculated for each step. Then, the axial stress of the same type of bolt A2 was measured by substituting the calculated energy attenuation into the estimation model. Repeating the measurement experiment under three different clamping lengths, the measurement results are shown in Table 6.

**Figure 12:**
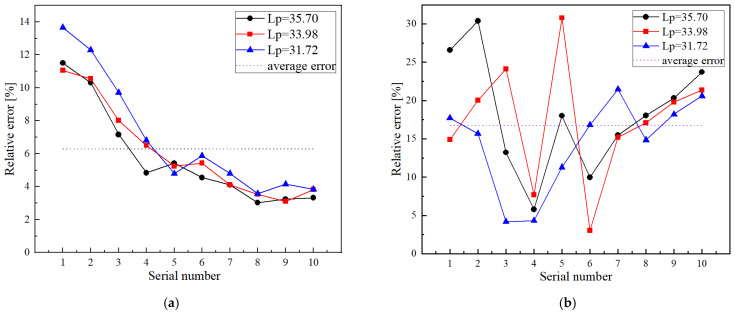
Relative error: (**a**) relative error of the proposed method; (**b**) relative error of the conventional acoustoelastic effect method.

Figure 12a shows the predicted relative error and the average relative error in the three clamping lengths. The average relative error is 6.28% and the variation trend of the predicted relative error in the three clamping lengths is relatively stable. It should be noted that the predicted results for the low-stress region (<80 MPa) have a larger error relative to the high-stress region (>80 MPa) in all clamping lengths. The relative errors of the high-stress region (>80 MPa) are less than 6%. However, the minimum relative error of the low region (<80 MPa) is 9.57%. On the one hand, the stress-dependent attenuation effect becomes weaker as the axial stress decreases. On the other hand, the nonlinearity of the stress distribution is enhanced in the low-stress region, which reduces the accuracy of the estimated model. For high-strength short bolts (working stress > 100 MPa), this method has a good measurement accuracy.

Axial stress measurement experiments based on the conventional acoustoelastic method were also conducted for the same three clamping lengths. Thirty sets of axial stresses were measured and the results are shown in Table 7. Compared with the three clamping length evaluation results of the two methods, the error band of the two methods can be observed in Figure 12. The average relative errors of the axial stress measured by the stress-dependent attenuation method and the conventional acoustoelastic effect method were 6.28% and 16.68%, respectively. Furthermore, the error bands of the predicted values were reduced significantly, from 8.8–5.9% of the attenuation method to 30.78–3.04% of the conventional method, which means that the measurement stability of the attenuation method is better.

Therefore, the attenuation energy ultrasonic method is more suitable for the axial stress measurement of high-strength short bolts, which makes the prediction results more accurate and stable. Furthermore, the following advantages of the stress-dependent attenuation method in practical applications give it better application prospects. First, compared with the conventional acoustoelastic effect, the stress-dependent attenuation effect is more sensitive to the stress changes, and the energy attenuation coefficient in the sensitive frequency band benefits from distinguishing the subtle changes of stress and improving the accuracy of prediction. Second, compared with the time-domain characterization, the spectrum analysis has better noise immunity. Third, the stress-dependent attenuation method does not need to measure the signal characteristics under zero stress, which improves the measurement efficiency and availability.

## 5. Conclusions

This study developed an axial stress measurement method for short bolts based on the stress-dependent scattering effect and energy attenuation coefficient. The estimation model of axial stress on the ultrasonic scattering attenuation is established based on the energy attenuation coefficient in the sensitive band. The energy attenuation coefficient in different frequency bands can show a more comprehensive analysis of attenuation in the frequency domain. Compared with other frequency bands, the axial stress estimation model based on the energy attenuation coefficient in the sensitive frequency band is more accurate. Ultrasonic experiments were performed with a magnetic transducer for the 45steel short bolt M10*54. The experimental results show that the energy attenuation coefficient in the sensitive band is well correlated with the axial stress of the bolt. Compared with the conventional acoustoelastic effect method based on the change of propagation time before and after bolt tightening, the proposed method has higher prediction accuracy and better stability. In addition, the method proposed in this article has not only been experimentally proven to be feasible for evaluating axial stresses in bolts but it is also applicable to validate some other similar shaft-like bars, and thus is well suited for structural health monitoring systems.

It is worth mentioning that the sensitive frequency band in this study is based on testing data acquired from the 45steel short bolt. The effective sensitive frequency band needs to be determined by the selected bolt material properties and transducer characteristics. Furthermore, the scattering assumption in this study is based on the equiaxial grain shape. When the ultrasonic wave passes through the material with irregular grain shapes, the model may produce large relative errors. In addition, the effect of non-uniform stress distribution on the estimation and the uncertainties in the model and experiment will be further investigated in future work.

## Figures and Tables

**Table 1:** Parameters of the used ultrasonic transducer.

Symbol	Parameter	Value
F0	Central frequency	10 MHz
a	Transducer radius	6.35 mm
Bw	−6 dB Bandwidth	5–13 MHz

**Table 2:** Parameters of the bolt specimens.

Name	Material	Length of Thread	Type
A1(M10 × 53.87)	C45	20.06 mm	Half-threaded
A2(M10 × 53.85)	C45	20.03 mm	Half-threaded

**Table 3:** Specimens’ material properties: density, ρ; bulk modulus, κ; second-and third-order single-crystal anisotropy coefficients, ν and η.

ρ	κ	ν	η
(kg/m^3^)	GPa	GPa	GPa
7800	166.67	−139	1666

**Table 4:** Prediction results in the sensitive and entire frequency bands.

Serial No.	Actual Stress	8.5–9.5 MHz *R*^2^ = 0.9951		5.5–10.5 MHz *R*^2^ = 0.9833	
		Predicted Value (MPa)	Relative Error (%)	Predicted Value (MPa)	Relative Error (%)
1	22.76	25.25	10.95	19.54	14.14
2	47.01	42.77	9.01	42.65	9.27
3	73.14	76.91	5.16	85.07	16.32
4	95.88	100.78	5.12	87.97	8.24
5	115.01	118.11	2.70	108.06	6.04
6	143.72	147.09	2.35	153.82	7.03
7	169.07	162.35	3.97	160.02	5.35
8	197.48	192.18	2.68	189.11	4.24

**Table 5:** Prediction results in the other four bands.

Serial No.	Actual Stress	5.5–6.5 MHz *R*^2^ = 0.8294		6.5–7.5 MHz *R*^2^ = 0.9330		7.5–8.5 MHz *R*^2^ = 0.9570		9.5–10.5 MHz *R*^2^ = 0.9601	
		Predicted Value (MPa)	Relative Error (%)	Predicted Value (MPa)	Relative Error (%)	Predicted Value (MPa)	Relative Error (%)	Predicted Value (MPa)	Relative Error (%)
1	22.76	32.21	41.55	18.58	18.33	20.14	11.49	13.59	40.25
2	47.01	61.03	29.83	44.19	5.99	41.22	12.3	55.04	17.10
3	73.14	110.82	51.53	82.21	12.40	80.21	9.66	69.74	4.64
4	95.88	63.04	34.25	88.08	8.13	89.98	6.15	108.86	13.54
5	115.01	113.42	1.38	101.87	11.42	120.71	4.96	129.04	12.20
6	143.72	181.60	26.36	134.98	6.08	135.93	5.42	152.45	6.08
7	169.07	152.70	9.68	160.75	4.92	162.08	4.13	164.15	2.91
8	197.48	175.01	11.38	206.28	4.46	204.41	3.51	206.62	4.63

**Table 6:** Measurement results of axial stress based on the energy attenuation coefficient method.

Serial No	1st Clamping Length *L_p_* = 35.70 (mm)			2nd Clamping Length *L_p_* = 33.98 (mm)			3rd Clamping Length *L_p_* = 31.72 (mm)		
	Actual Force (MPa)	Predicted Value (MPa)	Relative Error (%)	Actual Force (MPa)	Predicted Value (MPa)	Relative Error (%)	Actual Force (MPa)	Predicted Value (MPa)	Relative Error (%)
1	22.10	19.55	11.51	21.01	23.33	11.06	20.91	18.05	13.66
2	38.12	42.05	10.31	38.97	43.08	10.56	47.57	53.42	12.31
3	58.69	54.48	7.16	61.21	66.11	8.02	65.37	59.01	9.72
4	81.47	85.41	4.84	82.00	87.33	6.50	84.65	78.88	6.81
5	96.58	91.34	5.42	101.25	95.93	5.25	105.15	100.11	4.79
6	119.33	113.91	4.55	118.27	124.69	5.43	126.15	118.73	5.88
7	140.15	145.89	4.10	142.57	148.42	4.11	143.88	150.78	4.80
8	163.25	158.31	3.03	163.35	169.01	3.46	163.38	157.53	3.58
9	179.56	173.74	3.24	180.20	174.59	3.11	182.59	175.01	4.15
10	199.36	205.97	3.32	198.36	205.93	3.82	197.96	205.56	3.84

**Table 7:** Measurement results of axial stress based on the conventional acoustoelastic method.

Serial No	1st Clamping Length *L_p_* = 35.70 (mm)			2nd Clamping Length *L_p_* = 33.98 (mm)			3rd Clamping Length *L_p_* = 31.72 (mm)		
	Actual Force (MPa)	Predicted Value (MPa)	Relative Error (%)	Actual Force (MPa)	Predicted Value (MPa)	Relative Error (%)	Actual Force (MPa)	Predicted Value (MPa)	Relative Error (%)
1	22.34	28.27	26.58	21.03	24.16	14.89	21.18	24.93	17.71
2	40.19	52.39	30.36	38.60	46.34	20.06	41.20	47.66	15.68
3	59.43	51.57	13.21	63.56	48.22	24.12	66.67	63.88	4.18
4	81.14	76.43	5.80	81.80	75.52	7.67	83.28	79.67	4.33
5	102.32	120.75	18.02	102.78	134.42	30.79	105.08	116.94	11.29
6	121.67	109.55	9.96	123.94	120.16	3.04	125.51	104.38	16.83
7	138.22	159.61	15.48	137.83	158.78	15.20	142.11	172.62	21.47
8	162.04	191.26	18.04	162.31	190.04	17.09	160.33	184.12	14.84
9	183.66	220.96	20.31	185.03	221.655	19.79	181.75	214.84	18.21
10	198.93	246.11	23.72	196.12	238.04	21.37	198.21	239.038	20.60
